# Maternal knowledge, attitudes, and practices are associated with dental caries in preschool children in eastern United Arab Emirates

**DOI:** 10.3389/froh.2026.1706385

**Published:** 2026-02-18

**Authors:** Bothaina Alkaabi, Raghad Hashim, Raghavendra M. Shetty, Tarun Walia, Elias D. Berdouses

**Affiliations:** 1 Department of Clinical Sciences, College of Dentistry, Ajman University, Ajman, United Arab Emirates; 2 Department of Basic Medical & Dental Sciences, Centre of Medical and Bio-allied Health Sciences Research, College of Dentistry, Ajman University, Ajman, United Arab Emirates; 3 Department of Clinical Sciences, Center of Medical and Bio-allied Health Sciences Research, College of Dentistry, Ajman University, Ajman, United Arab Emirates

**Keywords:** attitude, caries experience, early childhood caries, knowledge, practice, UAE

## Abstract

**Objective:**

This study aims to investigate the relationship among knowledge, attitude, and related practices of mothers of preschool children toward their children's dental caries status in the Eastern Region of the United Arab Emirates (UAE).

**Methods:**

A cross-sectional study was conducted among 387 mothers of Emirati preschool children attending specialized dental centers in the Eastern Region of the UAE. Data were collected through direct interviews using a validated questionnaire to assess maternal knowledge, attitudes, and practices (KAP) related to their children’s oral health. Clinical oral examinations of the children were performed by a single calibrated examiner, following the World Health Organization (WHO) criteria.

**Results:**

The overall prevalence of dental caries among the examined children was 76.2%, with a mean decayed, missing, and filled teeth (dmft) score of 7.4. The majority of the mothers had a high level of knowledge (66.1%) and a positive attitude (89.7%) but a poor level of practice. Consequently, the overall KAP score was found to be moderate (48.1%) among nearly half of the participants. Higher maternal knowledge, attitude, and practice levels were associated with lower mean dmft among their children (*p* < 0.001).

**Conclusions:**

Maternal knowledge, attitudes, and practices (KAP) were important determinants of children's oral health.

## Introduction

Early childhood caries (ECC) remains one of the most prevalent chronic diseases affecting preschool children worldwide (1), despite being largely preventable. Global prevalence rates among children aged 4 years range from 12% to 98% (2), reflecting substantial variation across populations. In the United Arab Emirates (UAE), ECC continues to pose a significant public health challenge, with reported prevalence rates ranging between 41% and 83% among preschool children (3), even with the availability of advanced dental care services (2).

Mothers play a central role in shaping oral health behaviors during early childhood, a period when children are highly dependent on parental guidance (4). Maternal knowledge, attitudes, and practices (KAP) influence key determinants of ECC, including feeding behaviors, oral hygiene routines, fluoride use, and dental service utilization (5). Insufficient maternal knowledge and unfavorable practices have been associated with increased caries risk, whereas positive attitudes and appropriate oral health practices are linked to better oral health outcomes in children (6–8).

Previous studies have demonstrated a significant association between maternal oral health knowledge and children's dental caries experience. Systematic reviews (9) and observational studies (10, 11) indicate that children whose mothers possess higher levels of oral health knowledge and demonstrate positive oral health attitudes are less likely to develop dental caries. However, several studies report a discrepancy between maternal knowledge and actual oral health practices, suggesting that adequate knowledge does not always translate into appropriate behaviors (12).

Despite the established role of maternal knowledge, attitudes, and practices in shaping children's oral health, there is a lack of region-specific data on the relationship between maternal KAP and the oral health status of preschool-aged children in the UAE. Existing research primarily focuses on broad international populations, overlooking localized cultural, socioeconomic, and healthcare access factors that may influence oral health behaviors. Understanding this relationship is essential for developing targeted, mother-centered preventive strategies aimed at reducing early childhood caries. Hence, this study aims to assess knowledge, attitudes, and practices (KAP) of mothers of preschool children in the Eastern Region of the UAE and to examine their association with the caries experience of their children.

## Material and methods

A cross-sectional study was conducted between August and November 2024 among mothers of preschool-aged children attending specialized dental centers (SDCs) in the Eastern Region of the United Arab Emirates (UAE), which includes Fujairah, Khor-Fakkan, and Kalba. One SDC is located in each city. Mothers accompanying their preschool children during dental visits were invited to participate. Written informed consent was obtained from all participants after explanation of the study objectives. Participation was voluntary, and mothers were informed of their right to withdraw at any time. Ethical approval was obtained from the Research Ethics Committee of Ajman University and the Ministry of Health and Prevention.

A facility-based convenience sampling approach was used. All eligible mothers who accompanied their preschool-aged children to the selected specialized dental centers during the study period were consecutively invited to participate until the required sample size was achieved. Specialized dental centers were selected as recruitment sites because they provide comprehensive pediatric dental services and represent the primary point of oral healthcare access for preschool children in the Eastern Region of the UAE. The sample size was calculated using Cochran's formula for estimating a single population proportion, *n* = *Z*^2^ × *p*(1 − *p*)/*d*^2^. Assuming a caries prevalence of 50%, a 95% confidence level (*Z* = 1.96), and a margin of error of 5%, the minimum required sample size was 384 participants. To compensate for possible non-response or incomplete data, additional participants were recruited, resulting in an initial sample of 390 mothers. Three questionnaires were incomplete and excluded, yielding a final sample size of 387. Only mothers of medically fit Emirati children aged below 6 years were included in the study.

Data were collected using a structured questionnaire developed from validated WHO items and a previously published study (13, 14). The questionnaire was translated into Arabic using a forward–backward translation process to ensure linguistic and conceptual equivalence. In addition to linguistic translation, cultural adaptation was performed to ensure the questionnaire was appropriate for the local Emirati context. This included reviewing items for cultural relevance, clarity, and acceptability, particularly with respect to parental beliefs, child-rearing practices, and oral health behaviors. Feedback from the expert panel resulted in minor wording modifications to improve cultural appropriateness while preserving the original meaning of the items. Content validity was assessed by a panel of three pediatric dentistry experts, who reviewed the questionnaire for relevance, clarity, and appropriateness to the study objectives. Minor revisions were made based on their feedback. A pilot study involving 29 mothers from the target population was conducted to assess feasibility and clarity; pilot data were not included in the final analysis. The questionnaire was administered on-site at the specialized dental centers by the principal investigator through face-to-face interviews, ensuring completeness and clarification of participant queries. The questionnaire consisted of two sections: sociodemographic characteristics and 18 close-ended items assessing maternal knowledge, attitudes, and practices (KAP) related to their children's oral health.

To evaluate mothers’ knowledge of children's oral health, five questions were included. The participants received one point for each correct answer, while no points were awarded for incorrect responses. Similarly, evaluating mothers' practices regarding their children's oral health included seven questions, with scoring conducted in the same manner as the knowledge assessment. To evaluate mothers' attitudes toward their children's oral health, the participants were asked to agree or disagree with three statements. All questions in the questionnaire were close-ended. Attitudes were not classified as right or wrong but as positive or negative. Responses were scored as follows: “agree” received a score of 3, “don't agree” a score of 2, and “not sure” a score of 1. The total attitude score was calculated by summing these responses, with a score of 9 indicating agreement with all statements, reflecting a positive attitude. Conversely, a score of 6 indicated disagreement with all statements, and a score of 3 indicated not sure with all statements suggesting a negative attitude. Scores were categorized using Bloom's cutoffs (0%–59%, 60%–79%, and 80%–100%), where higher scores indicated better outcomes. The sum of each participant's knowledge, attitude, and practice scores was used to get their total KAP score.

After questionnaire completion, children underwent oral examination performed by the principal investigator in the dental clinic using standard dental diagnostic tools under dental chair illumination. Dental caries was assessed using the WHO criteria at the cavitation level, and the dmft index was recorded (13). Teeth with dental caries and restorations with recurrent caries were recorded as decayed (d). Teeth extracted due to caries were recorded as missing (m), and restored teeth, including teeth with crowns, were recorded as filled (f). The dmft score was calculated as the sum of decayed, missing, and filled teeth. The total time required to complete the questionnaire and oral examination for each child did not exceed 10 min, and approximately seven children were examined per day. Prior to data collection, intra- and inter-examiner calibration sessions were conducted with a second examiner. Examiner agreement was assessed using Cohen's kappa statistics, yielding values of 0.91 (intra-examiner) and 0.83 (inter-examiner), indicating almost perfect agreement.

Data were entered into Microsoft Excel and analyzed using R statistical software (version 4.4.3). Categorical variables were summarized as frequencies and percentages and analyzed using the chi-square test. Continuous variables were presented as means with standard deviations and 95% confidence intervals. Associations between maternal KAP scores and children's dmft were assessed using the Kruskal–Wallis test, followed by Dunn's *post hoc* test with Bonferroni adjustment. Regression analysis was made using a robust regression model. Statistical significance was set at *p* < 0.05.

## Results

The study included 387 children [175 (45.2%) males and 212 (54.8%) females]. The age of children varied, with 179 (46.3%) younger than 5 years and 208 (53.8%) aged 5 years. Over half of the mothers (56.3%) had a university degree. Employment was nearly balanced, with 53.5% employed and 46.5% unemployed. Almost two-thirds of the mothers were aged 30–39 years (61.8%). Most respondents (68.2%) reported a middle-income level. Regarding family size, 41.3% had 4–5 children. The overall prevalence of dental caries among the examined children was 76.2%, while the mean dmft (SD) for the group as a whole was 7.4 (5.1), which comprised 5.5 (4.9) decayed teeth, 0.5 (1.4) missing teeth, and 1.4 (2.6) filled teeth (data not presented).

Maternal education was significantly associated with mean dmft, with the children of mothers with higher education (6.6 ± 5.2) having significantly lower caries level than that of children of mothers with primary or high school degree (8.5 ± 4.8). The association with maternal occupation was also statistically significant, with the children of working mothers (6.7 ± 5.3) having significantly lower values than those of children of unemployed mothers (8.2 ± 4.7). In contrast, the association with the age of the mother, with mothers aged 40.50 years (8.0 ± 5.3) having the highest value. Additionally, there was no association with income level, with the children in families of mid-level income (7.6 ± 5.1) having higher values than those of children from low-income (7.3 ± 5.3) and high-income (6.4 ± 4.9) families. Finally, the association with the number of children was statistically significant, with cases in families having >5 children (9.0 ± 5.0) scoring significantly higher caries level than that of those in families having 1–3 children (7.0 ± 5.04). The association between family socioeconomic characteristics and their children's caries experience is presented in Table 1.

**Table 1:** Association between sociodemographic characteristics and dmft scores among children.

Parameter (*N* = 387)	*N*	%	dmft (mean ± SD)	*p*-value
Gender				
Male	175	45.2	−	−
Female	212	54.8		
Age of the child				
Less than 5 years	179	46.3	4.3 ± 1.0	
5 years	208	53.8		
Maternal education level				
Primary/high school	169	43.7	8.5 ± 4.8	<0.001*
University	218	56.3	6.6 ± 5.2	
Maternal professional status				
Unemployed	180	46.5	8.2 ± 4.7	0.002*
Employed	207	53.5	6.7 ± 5.3	
Age of the mother				
18–29 years	42	10.9	6.8 ± 5.3	0.378
30–39 years	239	61.8	7.2 ± 5.0	
40–50 years	106	27.4	8.0 ± 5.3	
Family income level				
Low	65	16.8	7.3 ± 5.3	0.312
Middle	264	68.2	7.6 ± 5.1	
High	58	15.0	6.4 ± 4.9	
Number of children in the family				
1–3	146	37.8	7.0 ± 5.0	0.016*
4–5	160	41.3	7.0 ± 5.1	
More than 5	81	20.9	9.0 ± 5.0	

* Statistically significant (*p* < 0.05).

The frequency distribution of mothers’ knowledge is presented in Table 2. The majority 83.5% believed that primary teeth should be preserved until they fall naturally. While 54.3% correctly identified age 6 as the time for the first permanent tooth eruption, others were either uncertain or had incorrect assumptions. When asked if bacteria from the mother's mouth can be passed to the child by using the same spoon for feeding, 302 respondents (78.0%) believed it could. Regarding fluoride in toothpaste, most participants (81.9%) recognized fluoride's role in preventing tooth decay. Nearly all (99.7%) correctly identified sugar as the main cause of tooth decay. The Frequency distribution of mothers' attitudes is presented in Table 3. The majority (88.63%) agreed that dental problems impact overall health. Almost three-quarters of the mothers supported the necessity of cleaning a child's teeth after meals, and a strong consensus (95.9%) favored parental assistance in tooth brushing until age 7.

**Table 2:** Frequency distribution of mothers’ knowledge.

Parameter (*N* = 387)	*N*	%
Should milk teeth be preserved until they fall naturally?		
Certainly	323	83.5
Don't know	29	7.5
Not necessarily	35	9.0
At what age does the first permanent tooth erupt in the oral cavity?		
At the age of 6 years	210	54.3
At the age of 8 years	83	21.5
I don't know	94	24.3
Do you think that bacteria (germs) from the mother's mouth can be passed to her child if she uses the same spoon when feeding her baby?		
Yes	302	78.0
No	32	8.3
I don't know	53	13.7
What is the role of fluoride?		
Prevents teeth decay	317	81.9
Prevents gum problems	15	3.9
Don't know	55	14.2
Which of the following nutrients leads to tooth decay?		
Sugar	386	99.7
Vegetables	0	0
Cheese	0	0
Meat	0	0
I don't know	1	0.3

**Table 3:** Frequency distribution of mothers’ attitude.

Parameter (*N* = 387)	Agree *N* (%)	Disagree *N* (%)	Not sure *N* (%)
Dental problems affect general health	343 (88.6)	23 (5.9)	21 (5.4)
It is necessary to clean the child's teeth after each meal	288 (74.4)	93 (24.0)	6 (1.6)
Parents should help and directly supervise the child brushing his/her teeth until he/she is 7 years old	371 (95.9)	7 (1.8)	9 (2.3)

Table 4 presents the result of mothers' practice. Not surprisingly, less than half (44.4%) of respondents began brushing their child's teeth when the first milk tooth appeared, while a substantial proportion (39.8%) delayed until all milk teeth had erupted. Additionally, almost half of the mothers (56.7%) brushed their child's teeth twice daily, and almost 71% of the mothers used fluoridated toothpaste for their children, while 58.1% reported changing their child's toothbrush every 2–3 months. Unfortunately, 46.5% of the children consume sugar between meals, and 60.5% of the mothers took their child to the dentist only when problems arise. Regarding the KAP score, the majority (66.1%) of the participants had a high level of knowledge, with a mean of 4.8 ± 1.3. The vast majority (89.7%) exhibited a positive attitude, with a mean of 8.5 ± 0.9. Most participants (87.6%) had poor practices, while 10.6% demonstrated a moderate level of practice, and only 1.8% had good practices, with a mean of 3.1 ± 1.3. This indicates a significant gap between knowledge, attitude, and actual practices. Finally, nearly half (48.1%) of the participants fell into the moderate KAP category, while 39.8% had a high KAP level and 12.1% low level. The mean KAP score was (16.4 ± 2.6) (data not presented).

**Table 4:** Frequency distribution of mothers’ practice.

Parameter	*N*	%
When did you start brushing your child's teeth?		
When the first milk tooth appears	172	44.4
When all milk teeth appear	154	39.8
I don't know/don't remember	46	11.9
I did not brush his/her teeth	15	3.9
How often do you brush your child's teeth?		
After each meal	28	7.5
Not regularly	68	18.3
Once daily	65	17.5
Twice daily	211	56.7
Does your child use toothpaste to clean his teeth?		
Yes	367	98.7
No	4	1.1
I don't know	1	0.3
Does your child's toothpaste contain fluoride?		
Yes	265	71.2
No	17	4.6
I don't know	90	24.2
When do you change your child's toothbrush?		
As soon as the brush bristles are bent	75	20.2
Every 2–3 months	216	58.1
Not regularly	81	21.8
What time does your child consume sugar?		
Between meals	180	46.5
Before bedtime	12	3.1
With meals	30	7.8
Not regularly	165	42.6
When do you take your child to visit the dentist?		
When problems appear	234	60.5
Every 6 months	67	17.3
Yearly	11	2.8
Not regularly	75	19.4

The association between caries experience and KAP scores is presented in Table 5. A significant association was observed between caries experience and all KAP scores for all variables (*p* < 0.001). Children who had a low mean dmft were more likely to have mothers with the highest knowledge score (9.3 ± 5.2). A similar pattern was observed in the attitude scores, with a mean dmft of 12.3 ± 6.6 in the low-caries group and 7.0 ± 5.0 in the high-caries group. Practice scores also showed a significant association (*p* < 0.001), being highest among children with low mean dmft (7.7 ± 5.1). For the overall KAP score, children with low caries level had a mean of 9.3 ± 5.5. Pairwise comparisons for all variables showed that mothers of children with a high risk of caries had significantly lower scores than those of mothers of children with lower risks (*p* < 0.001).

**Table 5:** Association between child's caries experience (dmft) and maternal knowledge, attitude, practice, and overall KAP scores.

Variable	Level	dmft (mean ± SD)	*p*-value
Knowledge	Low	9.3 ± 5.2	<0.001*
	Moderate	8.3 ± 4.6	
	High	6.7 ± 5.1	
Attitude	Low	12.3 ± 6.6	<0.001*
	Moderate	10.1 ± 4.4	
	High	7.0 ± 5.0	
Practice	Low	7.7 ± 5.1	<0.001*
	Moderate	5.6 ± 4.8	
	High	0.6 ± 1.5	
KAP	Low	9.3 ± 5.5	<0.001*
	Moderate	8.3 ± 4.7	
	High	5.8 ± 5.0	

* Statistically significant (*p* < 0.05).

In the regression model with dmft as the outcome presented in Table 6, a significant association was found with KAP score (*p* < 0.001), where higher KAP scores were associated with lower dmft levels. Family income level was also significantly associated (*p* = 0.018); children from middle-income families had higher dmft than that of those from low-income families. All other predictors were not statistically significant (*p* > 0.05).

**Table 6:** Multiple linear regression analysis predicting children's caries experience (dmft).

Parameter	Coefficient	95% confidence interval		Test statistic	*p*-value
		Lower	Upper		
KAP score	−0.5	−0.7	−0.2	−3.9	<0.001*
Maternal education level					
Primary/high school (*reference*)					
University	−0.4	−1.7	0.9	−0.6	0.575
Maternal professional status					
Unemployed (*reference*)					
Employed	−0.1	−1.3	1.2	−0.1	0.919
Age of the mother					
18–29 years (*reference*)					
30–39 years	−0.3	−2.3	1.7	−0.3	0.770
40–50 years	−0.3	−2.6	2.0	−0.3	0.792
Family income level					
Low (*reference*)					
Middle	2.0	0.3	3.6	2.4	0.018*
High	1.6	−0.5	3.6	1.5	0.141
Number of children					
1–3 (*reference*)					
4–5	0.3	−1.1	1.7	0.5	0.648
More than 5	0.4	−1.4	2.2	0.4	0.662

* Statistically significant (*p* < 0.05).

## Discussion

This study assessed maternal knowledge, attitudes, and practices regarding child oral health in the Eastern Region of the UAE and examined their association with children's caries experience. Despite the availability of free dental services for Emirati children, early childhood caries remains highly prevalent, consistent with previous regional reports (15, 16). These findings highlight persistent gaps in prevention that extend beyond access to care alone.

Importantly, this study contributes novel insight by demonstrating that high maternal awareness of oral health principles does not necessarily translate into preventive behaviors in a context where dental services are freely available. By examining maternal knowledge, attitudes, and practices alongside children's caries experience, the findings highlight a persistent gap between awareness and implementation, suggesting that access to care and knowledge alone are insufficient to reduce early childhood caries. This underscores the need for prevention strategies that prioritize behavioral reinforcement, anticipatory guidance, and sustained parental engagement rather than information delivery alone.

The burden of dental caries observed in this study aligns with findings from the UAE and neighboring countries (15–17). In the context of the identified gap between awareness and behavior, the high disease burden may reflect delayed presentation and symptom-driven dental attendance, as children included in this study were actively seeking dental treatment. These findings reinforce concerns that oral healthcare utilization in this population remains largely treatment-oriented rather than preventive, consistent with a previous report (14).

Mothers play a central role in shaping children's oral health behaviors through dietary regulation, oral hygiene supervision, and healthcare decision-making (18). In the present study, maternal education was associated with more favorable oral health outcomes in children, supporting evidence that higher educational attainment enhances oral health literacy and the adoption of preventive practices (14, 19). Similarly, children of employed mothers exhibited lower dmft scores, likely reflecting socioeconomic advantages that facilitate access to preventive care rather than differences in awareness alone, consistent with findings by Adil et al. (20). Maternal age did not demonstrate a consistent association with children's oral health outcomes, although children of older mothers tended to exhibit greater caries experience. This observation aligns with previous research suggesting that older mothers may have less exposure to updated preventive oral health information compared with younger mothers (6).

Contrary to common assumptions, family income did not show a clear linear relationship with caries experience. Children from middle-income households demonstrated greater caries experience than those from lower- or higher-income families, a pattern also reported by Chen et al. (21). This suggests that maternal education and family dynamics may exert a stronger influence on child oral health than income alone. Notably, children from larger families (having more than five children) had significantly higher dmft scores than those from smaller families, likely due to reduced parental supervision and competing caregiving demands, consistent with findings from a systematic review by Anwar et al. (22).

Although overall maternal knowledge regarding oral health was generally high, important gaps persisted. While most mothers recognized the importance of preserving primary teeth and understood the role of sugar in caries development, misconceptions remained regarding tooth eruption timelines and optimal preventive practices. Awareness of bacterial transmission from mother to child was higher than that reported in some previous studies (23), indicating improved understanding in this population; however, targeted education remains necessary to address remaining gaps.

Fluoride exposure is essential for caries prevention, and its safety and effectiveness are well established (5, 24). In the present study, fluoride awareness among mothers was generally consistent with prior studies reporting adequate knowledge of its preventive role (5, 25). Nevertheless, uncertainty regarding appropriate fluoride use persisted among a subset of participants, echoing findings from regional studies that highlight ongoing confusion surrounding fluoride exposure in young children (26). Such uncertainty may compromise effective caries prevention despite overall awareness.

Mothers play a key role in shaping their children's oral health behaviors through attitudes and role modeling (27). The present study demonstrated a generally positive maternal attitude toward oral health, with most mothers recognizing the impact of dental problems on overall health. Awareness of the importance of brushing after meals was high, exceeding what has been reported by Choufani and Barakat (14). In addition, most mothers supported continued parental supervision of toothbrushing until the age of seven, consistent with recommendations that young children lack the motivation and manual dexterity to brush effectively on their own (28). Although most mothers reported using fluoridated toothpaste, one-quarter were uncertain about its fluoride content, revealing a knowledge gap that may compromise caries prevention.

Despite high awareness of sugar as a risk factor, almost half of mothers provided sugary snacks between meals, consistent with previous studies (5, 14), although contrasting findings have been reported elsewhere (4). In addition, approximately two-thirds of respondents reported visiting the dentist only when dental problems occurred, in line with existing literature (29), indicating limited utilization of preventive dental services. Delayed initiation of toothbrushing, inconsistent brushing routines, frequent provision of sugary snacks, and symptom-driven dental visits were commonly reported, reflecting a persistent gap between knowledge and behavior (5, 14, 29).

The observed association between maternal KAP scores and children's caries experience suggests that higher levels of maternal knowledge, attitudes, and practices are associated with lower dmft, reinforcing the role of parental behavior as a modifiable determinant of early childhood caries. The association between family income and dmft was also notable, with higher caries levels observed among children from middle-income families compared with those from low-income households. This finding contrasts with previous reports indicating greater caries risk among low-income children (30) and may reflect context-specific factors within the studied population, such as dietary habits, caregiving practices, or patterns of dental service utilization.

In the UAE, one of the key challenges in addressing early childhood caries is limited maternal awareness regarding the importance of early dental care. The widespread perception that primary teeth are less important than permanent teeth often leads to delayed dental visits and inadequate preventive practices (17), contributing to the persistence of untreated caries. These findings highlight the need for early, proactive interventions that emphasize the significance of primary dentition and the role of maternal behaviors in shaping children's oral health outcomes.

At a broader level, preventive strategies should prioritize anticipatory guidance beginning in early childhood, including establishment of a dental home, initiation of dental visits by the age of one, and counseling on oral hygiene practices, fluoride use, and diet (31). Community-based and policy-driven interventions integrating oral health into primary care and maternal–child health services may further strengthen prevention efforts (32, 33). Interdisciplinary collaboration among dental professionals, pediatricians, educators, and community health workers is essential to improve outreach and continuity of care (34). Importantly, culturally adapted oral health education that addresses local beliefs and practices may enhance the effectiveness and acceptance of these interventions within the UAE context.

Limitations should be acknowledged. The use of self-reported questionnaires may introduce social desirability bias, potentially leading to overestimation of positive behaviors (35). In addition, the conversion of attitudinal responses into numerical scores may oversimplify complex constructs and should therefore be interpreted as general trends rather than precise measures. Furthermore, dental caries were assessed using the WHO diagnostic criteria, which do not account for early non-cavitated lesions; this may have resulted in an underestimation of the true caries prevalence. The cross-sectional design of the study also limits the ability to establish temporal or causal relationships between variables. Finally, the focus on Emirati mothers attending government dental centers in a single region limits the generalizability of the findings to other populations and care settings.

In conclusion, this study confirms that early childhood caries remains highly prevalent among Emirati children despite free access to dental services, indicating that availability of care and maternal awareness alone are insufficient to prevent disease. Although maternal knowledge and attitudes toward child oral health were generally high, a persistent gap between awareness and preventive practices was observed and was reflected in children's caries experience. Maternal education, employment status, and family size were important determinants of oral health outcomes, whereas income alone showed no consistent protective effect. These findings highlight the need for prevention strategies that move beyond information provision toward behavior-focused interventions, anticipatory guidance, and sustained parental engagement. Integrating culturally appropriate oral health promotion into maternal and child health services may be essential to reducing the burden of early childhood caries in the UAE.

## Clinical relevance

Maternal behaviors play a critical role in shaping early childhood oral health outcomes. The findings of this study indicate that high maternal knowledge and positive attitudes alone may be insufficient to prevent early childhood caries if not accompanied by consistent preventive practices. For clinicians, this underscores the importance of incorporating behavior-focused counseling, anticipatory guidance, and sustained parental engagement into routine pediatric dental care. Early identification of gaps between knowledge and practice may support more effective prevention strategies and contribute to reducing the burden of early childhood caries.

## Data Availability

The raw data supporting the conclusions of this article will be made available by the authors, without undue reservation.
